# Deoxypodophyllotoxin Inhibits Cell Growth and Induces Apoptosis by Blocking EGFR and *MET* in Gefitinib-Resistant Non-Small Cell Lung Cancer

**DOI:** 10.4014/jmb.2101.01029

**Published:** 2021-03-05

**Authors:** Han Sol Kim, Ha-Na Oh, Ah-Won Kwak, Eunae Kim, Mee-Hyun Lee, Ji-Hye Seo, Seung-Sik Cho, Goo Yoon, Jung-Il Chae, Jung-Hyun Shim

**Affiliations:** 1 Department of Pharmacy, College of Pharmacy, Mokpo National University, Jeonnam 58554, Republic of Korea; 2 College of Pharmacy, Chosun University, Gwangju 61452, Republic of Korea; 3 College of Korean Medicine, Dongshin University, Naju, Jeonnam 58245, Republic of Korea; 4 Department of Dental Pharmacology, School of Dentistry, Jeonbuk National University, Jeonju 54896, Republic of Korea; 5 The China-US (Henan) Hormel Cancer Institute, Zhengzhou, Henan 450008, P.R. China; 6 Department of Biomedicine, Health and Life Convergence Sciences, BK21 Four, Biomedical and Healthcare Research Institute, Mokpo National University, Jeonnam 58554, Republic of Korea

**Keywords:** Deoxypodophyllotoxin, gefitinib-resistant, lung cancer, apoptosis

## Abstract

As one of the major types of lung cancer, non-small cell lung cancer (NSCLC) accounts for the majority of cancer-related deaths worldwide. Treatments for NSCLC includes surgery, chemotherapy, and targeted therapy. Among the targeted therapies, resistance to inhibitors of the epidermal growth factor receptor (EGFR) is common and remains a problem to be solved. *MET* (hepatocyte growth factor receptor) amplification is one of the major causes of EGFR-tyrosine kinase inhibitor (TKI) resistance. Therefore, there exists a need to find new and more efficacious therapies. Deoxypodophyllotoxin (DPT) extracted from *Anthriscus sylvestris* roots exhibits various pharmacological activities including anti-inflammation and anti-cancer effects. In this study we sought to determine the anti-cancer effects of DPT on HCC827GR cells, which are resistant to gefitinib (EGFR-TKI) due to regulation of EGFR and MET and their related signaling pathways. To identify the direct binding of DPT to EGFR and MET, we performed pull-down, ATP-binding, and kinase assays. DPT exhibited competitive binding with ATP against the network kinases EGFR and MET and reduced their activities. Also, DPT suppressed the expression of p-EGFR and p-MET as well as their downstreat proteins p-ErbB3, p-AKT, and p-ERK. The treatment of HCC827GR cells with DPT induced high ROS generation that led to endoplasmic-reticulum stress. Accordingly, loss of mitochondrial membrane potential and apoptosis by multi-caspase activation were observed. In conclusion, these results demonstrate the apoptotic effects of DPT on HCC827GR cells and signify the potential of DPT to serve as an adjuvant anti-cancer drug by simultaneously inhibiting EGFR and MET.

## Introduction

Lung cancer is one of the most common malignant cancers all over the world [1]. It is the leading cause of cancer-related death in men and the 5-year survival rate is only 16% [2, 3]. Lung cancer is divided into two subgroups, small-cell lung cancer (SCLC, 15%) and non-small cell lung cancer (NSCLC, 85%), both of which are treated via different therapeutic methods [4]. Especially, NSCLC treatment includes tumor radical resection, chemotherapy, targeted therapy, and immunotherapy [5].

The first-generation reversible epidermal growth factor receptor (EGFR) inhibitors, gefitinib (GEF) and erlotinib are the anti-cancer drugs that target EGFR. Afatinib and dacomitinib are classified as second-generation irreversible EGFR inhibitors. Recently, a third-generation EGFR inhibitor, osimertinib, has been approved by the FDA based on its superiority to GEF and erlotinib. EGFR-tyrosine kinase inhibitor (TKI) GEF is well-established for the treatment of *EGFR* gene mutation-positive NSCLC [6]. *EGFR* gene mutations are present in the tyrosine kinase domain such as exon 19 deletions (44%), L858R mutations (41%), and other rare mutations [7]. GEF has been reported as effective and selective chemotherapy; however, patients gradually acquired resistance to GEF within several months of treatment. It was found that *MET* amplification is the main mechanism of resistance to third-generation EGFR-TKIs as well as GEF [8]. Therefore, we used the HCC827GR cell line that contained *MET* amplification. Consequently, patients who had been using TKI had poor overall survival; consequently, effective treatment is required to overcome the resistance to EGFR-TKIs [9].

**Fig. 1:**
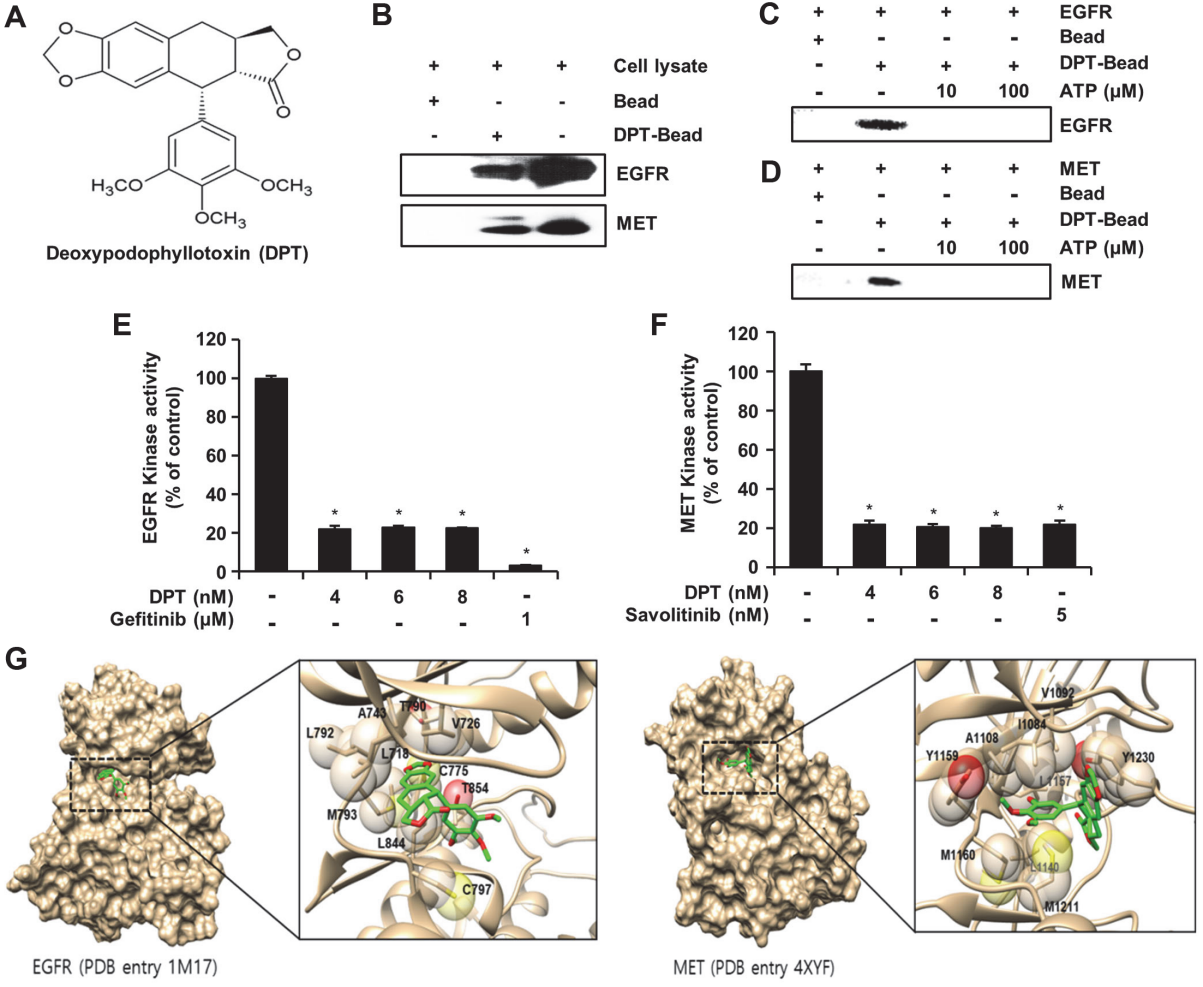
Deoxypodophyllotoxin (DPT) directly interacts with EGFR or MET. (**A**) Structure of DPT. (**B**) Pull down assay. Lysate from HCC827GR cells was incubated with Sepharose 4B-only beads and Sepharose 4B-conjugated DPT and the experiment was administered by described under “Material and methods”. Sepharose 4B beads; lane 1, DPT- Sepharose 4B beads; lane 2, Input control; lane 3, EGFR or MET was immunoprecipitated using DPT-Sepharose 4B beads that DPT binds EGFR or MET *ex vivo*. (**C**) Active EGFR (100 ng) or (**D**) MET (100 ng) was incubated with ATP at various concentrations (0, 10, 100 μM) and DPT-Sepharose 4B or Sepharose 4B. EGFR or MET was bound to DPT competitively with ATP. The pulled-down proteins were confirmed by western blotting. (**E**) EGFR or (**F**) MET kinase activity of DPT by ADP-Glo kinase assay. Each experiment was done in triplicate independently, and data represent the mean value ± SD (*n* = 3). * *p* < 0.05. (**G**) A predicted binding pose of DPT for EGFR (left) and MET (right). The receptor for EGFR and MET was available as PDB entry 1M17 and 4XYF, respectively. As shown as the surface representation of the receptor, DPT (stick) was tied in ATP binding and the ligandreceptor interaction was zoomed in details. The transparent sphere representation indicated the hydrophobic sidechain of ATP binding pocket related on the hydrophobic interactions. Notably, the complex of EGFR and MET was stabilized by the hydrophobic interactions.

Deoxypodophyllotoxin (DPT) is a naturally occurring flavonoid in *Anthriscus sylvestris* roots and its chemical name is (5S,5aS,8aS)-5-(3,4,5-trimethoxyphenyl)-5a,8,8a,9-tetrahydro-5H-[2]benzofuro[5,6-f][1,3]benzodioxol-6-one (Fig. 1A). DPT is an analog of podophyllotoxin and has been studied extensively for various pharmacological activities including anti-inflammatory, anti-viral [10], anti-proliferative, anti-platelet aggregation, and liver protection [11]. Previously it has been reported that DPT inhibited cell growth of cholangiocarcinoma, breast cancer, glioblastoma, and gastric cancer [12]. However, there are no studies on the anti-cancer effect of DPT on gefitinib-resistant lung cancer cells.

Anti-cancer drugs destroy cancer cells through apoptosis and cell cycle arrest. Apoptosis is a genetically controlled cell death mechanism that is important for various biological processes [13]. Apoptosis is morphologically featured by cell shrinkage, membrane blebbing, chromosomal DNA fragmentation, chromatin condensation, nuclear fragmentation, and formation of apoptotic bodies [14]. While cell cycle processes are related to cell growth, it is important to regulate cell cycle progression. Two main checkpoints of cell cycle processes are the G2/M checkpoint and G1/S checkpoint [15]. G2/M checkpoint of the cell cycle is controlled by maturation promoting factors, including cyclin-dependent kinase 1 (Cdk1) and cyclin B1 [16]. Through the G2/M arrest, the Cdk1/cyclin B1 complex is altered, which further leads to incomplete mitosis which may induce apoptosis [17].

The purpose of this study was to investigate whether DPT could prevent cell proliferation through EGFR and MET suppression using human gefitinib-resistant NSCLC cells. We found that DPT reduced the kinase activity of EGFR and MET, and inhibited both EGFR and MET downstream pathway. To investigate the underlying mechanism of DPT induced apoptosis, colony formation, cell cycle distribution, reactive oxygen species (ROS) production, mitochondrial membrane potential (MMP) depolarization, multi-caspase assays, and western blotting were performed. The results clarify the action mechanism of DPT in NSCLC and suggest that DPT could be a potential agent for the treatment of TKI-resistant lung cancer.

## Material and Methods

### Reagents and Antibodies

The synthesis method of DPT has been described in a previous report [18]. DPT was dissolved in dimethyl sulfoxide (DMSO) from Sigma Chemical Company (USA). GEF was obtained from Cayman Chemical (USA). RPMI-1640 medium, phosphate buffered saline (PBS), fetal bovine serum (FBS), L-glutamate and trypsin, penicillin and streptomycin were purchased from Hyclone (USA). The primary antibodies against Actin, cyclin B1, cdc2, p21, GRP78, CCAAT/enhancer-binding protein homologous protein (CHOP), death receptor (DR)4, DR5, Bid, Bcl-xl, Mcl-1, Bad, cytochrome C (cyto C), α-tubulin, COX4, apoptotic protease activating factor-1 (Apaf-1), poly (ADP-Ribose) Polymerase (PARP) and ErbB3 were purchased from Santa Cruz Biotechnology (USA). Antibodies to phosphor(p)-EGFR (Tyr1068), EGFR, p-AKT (Ser473), AKT, p-ERK (Thr202/Tyr204), ERK, p-ErbB3 (Tyr1289), p-MET (Tyr1234/1235) and MET were obtained from Cell signaling Technology (USA).

### Cell Culture

HCC827GR (Gefitinib-resistant and *MET*-amplified HCC827) cells were willingly provided by Professor Pasi A. Jänne, Department of Medical Oncology, Dana-Farber Cancer Institute, Boston, MA, USA [19]. HCC827GR cells were cultured in RPMI-1640 supplemented with 10% FBS and 100 U/ml penicillin-streptomycin and incubated at 37°C with 5% CO_2_.

### Western Blotting

Cells treated with DPT or GEF were suspended by RIPA buffer (iNtRON biotechnology, Korea) and incubated for 10 min on ice. Then each sample was sonicated in the ice. The DC Protein Assay (Bio-RAD, USA) was used to quantify the lysate protein concentrations. The same amounts of cellular lysates taken from each sample was separated by 8, 10 or 15% sodium dodecyl sulfate-polyacrylamide gel electrophoresis (SDS-PAGE). The proteins were transferred to polyvinylidene fluoride membranes (Merck Millipore, USA) and were blocked with 3% or 5%(w/v) skim milk in PBST (PBS with 0.1% Tween 20) for 2 h at room temperature (RT). After washing three times the membranes with PBST for 10 min, respectively, the membranes were incubated with indicated primary antibodies (1:1000) at 4°C overnight. Following 30 min washing, the blots were incubated with the consistent horseradish peroxidase-conjugated secondary antibodies (1:5000). The labeled proteins were visualized using an ImageQuant LAS 500 (GE Healthcare, Sweden) with Western blotting luminal reagent (USA).

### Pull-Down Assay

To identify direct interaction between DPT and EGFR or MET, HCC827GR cell lysates were mixed with Sepharose 4B beads or DPT conjugated-Sepharose 4B beads in reaction buffer containing 5 mM EDTA, 50 mM Tris (pH 7.5), 150 mM NaCl, 1 mM/l dithiothreitol, 2 μg/ml bovine serum albumin, 0.01% Nonidet P-40, 0.02 mM phenylmethylsulfonyl fluoride and 1X proteinase inhibitor. After incubation with moderate rocking overnight at 4°C, the beads were rinsed out six times with a washing buffer. The proteins bound to the beads were visualized by Western blotting with EGFR and MET antibodies.

### ATP-Competitive Binding Assay

Recombinant active EGFR or MET (100 ng) and indicated concentrations of DPT were pre-incubated at 4°C for 2 h. Then Sepharose 4B (negative control) or DPT conjugated-Sepharose 4B beads were added the mixture. The beads were incubated at 4°C overnight and then washed six times with washing buffer. The protein bound to the beads were analyzed by Western blotting.

### Kinase Assay

EGFR and MET kinase activities were identified using the EGFR (#3831) and MET (#3361) active kinase enzyme systems (Promega, USA) and ADP-Glo kinase assay kit (Promega), each. The EGFR (1.8 ng/μl) and MET (7 ng/μl) were responded in a 384-well plate with DPT (4, 6 or 8 nM) or 1 μM of GEF or 5 nM of savolitinib, 0.2 μg/μl of substrates, 5 μM or 10 μM of ATP and kinase reaction buffer including 0.1 mg/ml BSA, 50 μM DTT, 20 mM MgCl_2_, 2 mM MnCl_2_, 100 μM sodium vanadate and 40 mM Tris (PH 7.5) at RT for 1 h. 5 μl of ADP-Glo reagent (ADP-Glo kinase assay kit; Promega) was added to all wells to deplete the remaining ATP and complete the kinase reaction and reacted at RT for 40 min. Each well of the 384-well plate was added with 10 μl of kinase detection reagent. Luminescence reaction was detected with a Centro LB 960 microplate luminometer (Berthold Technologies, Germany) for 0.5 s.

### Molecular Modeling and Simulation

To investigate a possible binding pose of two receptor tyrosine kinases, EGFR and MET, a molecular docking simulation was performed using Autodock Vina software. The three- dimensional (3D) X-ray structures were downloaded for EGFR with erlotinib (PDB entry 1M17) and MET with quinoline analogue (PDB entry 4XYF). The 3D structure of DPT was built by Marvin sketch software. To search possible bind modes efficiently, the protein-ligand binding site should be defined including ATP binding site and the nucleotide binding site. The active sites were assigned as Val745, Asp855, Leu718-Val726, and Thr790-Gln791 for EGFR and Lys1110, and Ile1084-Val1092 for MET. According to the score of binding affinity computed by Autodock Vina, the top three binding poses were selected by less than 0.5 kcal/mol of the difference of the score value. The three possible complex poses were run using molecular dynamics (MD) simulation to confirm the thermal stability. MD could produce to be similar to the experimental environment such as a physiological condition (310 K and 1 atm) and aqua solvent environment and overcome the lack of a rigid docking simulation. The docking complex was solvated by TIP3P water model and neutralized by adding ions. Amber14SB force field (ff14SB) and general amber force field (GAFF) were applied for the kinase receptor and the ligand, respectively. Pre-equilibrium of isothermal-isobaric (NPT) condition runs for 500 ps and then canonical (NVT) ensemble was equilibrated for 100 ns. The simulation temperature and pressure were at 310 K and 1 atm, respectively. The timestep was about 2 fs using fixing a bond including a hydrogen atom. Periodic boundary condition was applied using Verlet cut-off scheme. The cut-off of a non-bonding force was set up about 9.0 Å. For the data analysis, the converged data such as steady time profile of MD simulation was used for the last 50 ns. MD simulation was performed using Gromacs software.

### MTT Assay

HCC827GR cells were seeded on 96-well plates and incubated at 37°C, CO_2_ incubator, overnight, then treated with various concentrations of DPT or GEF for 24 h and 48 h. 3-(4,5-Dimethyl-2-thiazolyl)−2,5-diphenyl-2H-tetrazolium bromide (MTT) was added to each well and incubated at 37°C for 2 h then 100 μl of DMSO was added to dissolve the formazan. Absorbance of samples was measured at 570 nm by a multiscan GO spectrophotometer (Thermo Scientific, Finland). Cell survival rate was calculated by (OD570 nm of the drug-treated well - OD570 nm of the blank well)/(OD570nm of the non-treated well - OD570 nm of the blank well) × 100%.

### Anchorage-Independent Cell Growth Assay

Layers of 3 ml of 0.6% agarose in complete medium (BME, 10% FBS, 5 μl gentamicin and 2 mM L-glutamine) were prepared in 6-well plates. Cells were suspended in 1 ml of 0.3% agarose in complete medium and overlaid on top of the bottom layer agar. Both layers were treated different concentrations of DPT or 1 μM of GEF. The agar plates were incubated at 37°C, CO_2_ incubator for 2 weeks. The number of colonies were observed using microscope (Leica Microsystem, Germany).

### Annexin V/7-Aminoactinomycin D (7-AAD) Staining

The apoptosis measurement was assessed by Annexin V/7-aminoactinomycin D (7-AAD) stained cells using a Muse Annexin V & Dead Cell Kit (MCH100105, Merck Millipore, USA). HCC827GR (1.65 × 10^5^ cells/well) cells were seeded into 6-well plates and treated with different concentrations (6, 8 nM) of DPT, 1 μM of GEF or DMSO for 48 h. Cells were harvested and added to 100 μl of Muse Annexin V & Dead Cell reagents. The cells and reagents were mildly mixed and incubated in dark condition at RT for 20 min. Fluorescence of Annexin V/7-AAD treated cells was detected by Muse Cell Analyzer (Merck Millipore).

### Cell Cycle Analysis

The cell cycle analysis was carried out as instructed in the Muse Cell Cycle Kit (MCH100106, Merck Millipore) according to the manufacturer’s instructions. For cell cycle analysis, HCC827GR (1.65 × 10^5^ cells/well) cells were seeded into 6-well plates and treated with various concentration of chemicals for 48 h. Cells were harvested by trypsin treatment and washed three times with PBS. Then obtained samples were fixed in 70% cold ethanol at -20°C. Ethanol-fixed cells were centrifuged at 4°C, 4,000 rpm for 10 min and the supernatant was discarded. After washing with PBS, cell pellets were resuspended with 200 μl of Muse Cell Cycle Reagent in the dark for 30 min at RT. After staining, the DNA contents were processed for cell cycle analysis using Muse Cell analyzer. The experiments were performed in triplicate.

### ROS Measurements

The measurement of oxidative stress cells was quantified by Muse Oxidative Stress Kit (MCH100111, Merck Millipore). The cells were seeded in 6-well plates overnight and then treated with DPT (0, 6, or 8 nM) or 1 μM GEF for 48 h. Cells were harvested and washed with assay buffer, and then incubated with Muse Oxidative Stress Reagent working solution in the dark at 37°C for 30 min. The percentage of cells undergoing oxidative stress were identified using the Muse Cell Analyzer and Muse analysis software (Merck Millipore).

### Mitochondria Membrane Potential (MMP) Assay

Cells (1.65 × 10^5^ cells/well) were seeded into 6-well plate and stabilized at 37°C, CO_2_ incubator for 24 h. Then the cells were treated with DPT (0, 6 or 8 nM) or 1 μM GEF, and incubated at 37°C, CO_2_ incubator for 48 h. Cells were harvested and washed with assay buffer then stained with the Muse Mitopotential working solution (MCH100110, Merck Millipore) at 37°C for 20 min. After then 7-AAD was added and incubated for 5 min at RT. MMPs were measured with the Muse Cell Analyzer.

### Isolation of Cytosol and Mitochondria Fractionation

HCC827GR cells were treated with different concentrations of DPT or GEF and collected using a cell scrapper then washed with PBS at 4°C. The harvested cells were suspended with plasma membrane extraction buffer [0.01 mg/ml aprotinin, 1 mM EDTA, 1 mM EGTA, 10 mM HEPES (pH 8.0), 10 mM KCl, 0.01 mg/ml leupeptin, 1.5 mM MgCl_2_∙6H_2_O, 0.1 mM phenylmethylsulfonyl fluoride, 250 mM sucrose] and added 0.1% of digitonin then powerfully vortexed for 1 min. The samples were centrifuged at 13,000 rpm for 30 min at 4°C to acquire the cytosolic fraction. Remnants containing mitochondria were washed by the plasma membrane extraction buffer. Then, 0.5% of triton X-100 was added and the sample was centrifuged at 13,000 rpm for 30 min at 4°C. Hence, the supernatant included the mitochondrial fraction.

### Multi-Caspase Assay

Multi-Caspase was performed utilizing a Muse Multi-Caspase Kit (MCH100109, Merck Millipore) following the manufacturer’s instructions. A total of 1.65 × 10^5^ cells/well were seeded per 6-well plate and cultured with different concentration of DPT or GEF for 48 h. The cells were harvested to assess quantitative caspase activity. The cell pellets suspended with Muse Multi-Caspase reagent working solution were reacted at 37°C for 30 min. Then 7‐AAD working solution was stained to each sample and the cells were analyzed by a Muse Cell analyzer.

### Statistical Analysis

Statistical analysis was performed using the software GraphPad Prism statistics (v5, GraphPad Software, USA, RRID: SCR_002798). Statistical comparisons of all data were analyzed using one-way or two-way analysis of variance (ANOVA) followed by Dunnett’s post hoc test and expressed as mean ± standard deviation (SD). *p* values <0.05 was regarded as statistically significant.

## Results

### Interaction of DPT with EGFR and MET in HCC827GR Cells

To elucidate the direct binding of DPT with EGFR or MET, *ex vivo* pull-down assays with DPT-Sepharose 4B or Sepharose 4B beads were performed. Incubation of HCC827GR cell lysate with DPT-Sepharose 4B or Sepharose 4B beads followed by pull-down and Western blot analysis revealed direct interaction of DPT with EGFR and MET (Fig. 1B). To further investigate how DPT binds to EGFR and MET, we conducted a pull-down assay with 10 or 100 μM concentration of ATP. Change in the binding ability of DPT to EGFR and MET was observed in response to an increase in the dose of ATP. Results revealed that DPT is an ATP-competitive inhibitor that decreases EGFR and MET kinase activities (Figs. 1C and 1D). The effect of DPT on EGFR or MET kinase activity was explained using in vitro kinase assay. Kinase assay data showed that DPT significantly suppressed EGFR (Fig. 1E) and MET (Fig. 1F) activities similar to the positive control (GEF and savolitinib). In the results of previous papers treated with savolitinib in HCC827GR cells [20], the IC_50_ of savolitinib was 1.1 nM. These results suggest that DPT inhibits the kinase activity of EGFR and MET in an ATP-competitive manner. Fig. 1G shows the predicted binding pose of DPT for EGFR and MET. As can be seen in the docking complex of EGFR and MET, DPT is stuck tightly in the ATP-binding pocket of the catalytic domain. The main conformational stability was driven by non-bonded contacts such as hydrophobic interactions. In EGFR, the tetra ring part (A-D rings) of DPT lay on Met793 of the hinge (residues 788-797) and was surrounded by the hydrophobic side chains such as Leu718 and Val726 of the nucleic acid binding site (residues 712-731), Leu844 and Cys775. In addition, DPT reached the gatekeeper Thr790 which determined the depth of the ligand pocket. Relatively, the 3’,4’,5’-trimethoxy phenyl ring (E ring) demonstrated contact with Thr854 and was partially exposed to the solvent environment. In the complex of MET, the A-D rings of DPT relied upon p-p interaction with Tyr 1230 and faced the activation loop (1231-1244). The E ring of DPT was sandwiched between the hydrophobic side chains of the two groups. One group like the upper cover was composed of Ile1084, Val1092, Ala1108 of P-loop, and Tyr1159 of the hinge region. The other group like the lower cover was made of Met1160, Leu1140, and Met1211. In addition, one methoxy group of E ring was close to Leu1157 deep inside. Considering the most stable poses for EGFR and MET, DPT was identified to be well suitable to the ATP binding pocket and the ligand-protein interaction was notably related in the hydrophobic interactions. DPT occupied the catalytic domain of the cytoplasm and the activator ATP was subsequently blocked. These results were consistent with the experimental results and it is hypothesized that DPT could be a potent dual-acting inhibitor for EGFR and MET as an ATP-competitive compound.

### DPT Regulates EGFR and MET Signaling Pathway

**Fig. 2:**
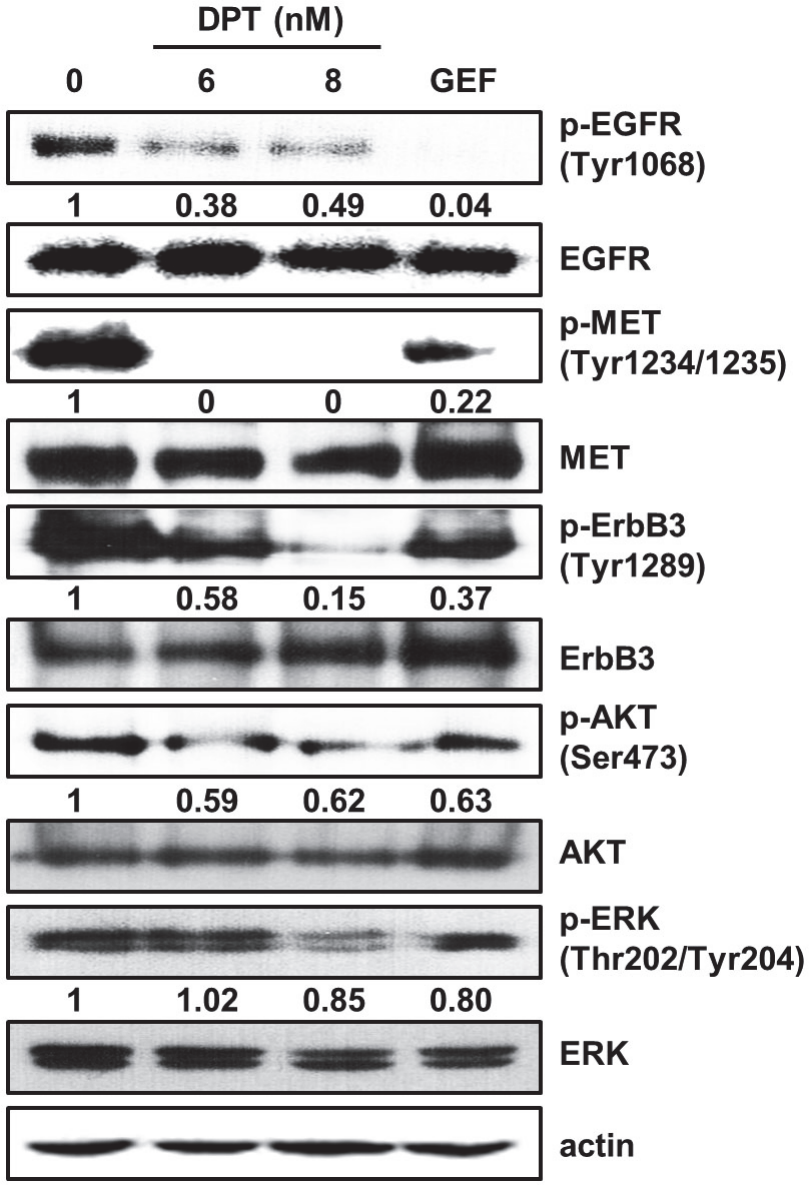
Effects of DPT on EGFR or MET signaling pathways in HCC827GR cells. Cells were treated with DPT or GEF for 48 h. The phosphorylated and total protein expression were evaluated by Western blot; p-EGFR (Tyr1068), EGFR, p-MET (Tyr1234/1235), MET, p-ErbB3 (Tyr1289), ErbB3, p-AKT (Ser473), AKT, p-ERK(Thr202/Tyr204), ERK and actin.

To explore whether the inhibitory effects of DPT on EGFR and MET kinase activities affect EGFR and MET signaling pathway, Western blotting was employed to identify the effects of DPT on phosphorylation and expression levels of EGFR, MET, ErbB3, AKT, and ERK (Fig. 2). Phosphorylated levels of EGFR, MET, and ErbB3 were decreased in response to DPT treatment compared with their total expression levels. AKT and ERK are crucial kinases in the RTK downstream pathways. The investigation of phosphorylation and expression levels of these proteins revealed a decrease compared with their total levels. GEF failed to suppress the above signaling cascades despite inhibiting p-EGFR expression. These results suggest that DPT is a potent EGFR and MET kinase inhibitor and regulates EGFR and MET downstream signaling pathways (Fig. 2).

### DPT Suppresses the Growth of Lung Cancer Cells

**Fig. 3:**
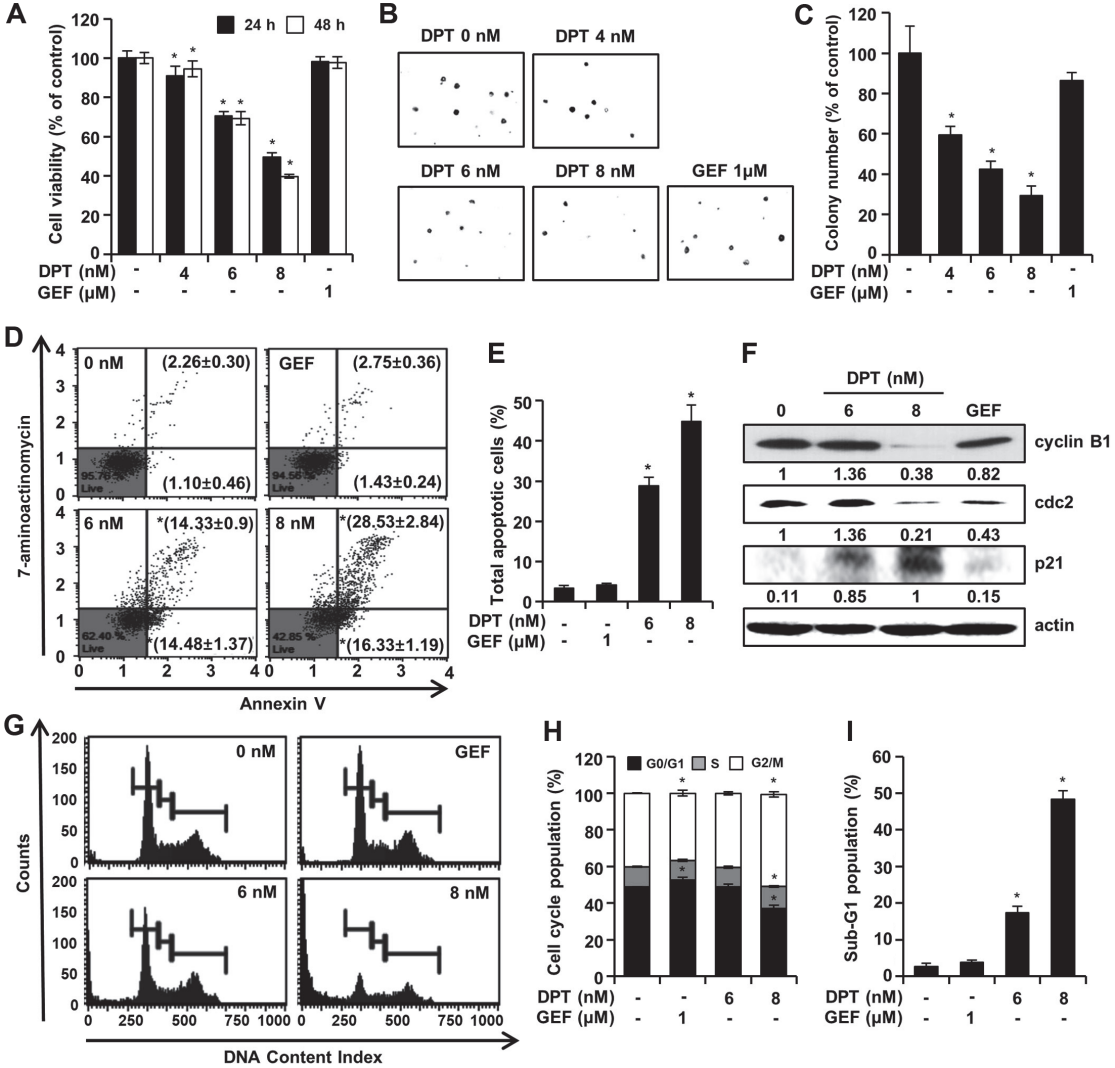
DPT inhibits cell viability and induces apoptosis and cell cycle arrest in HCC827GR cells. (**A**) MTT assay shows the effect of DPT on HCC827GR cell viability. Cells were treated with varying concentration of chemicals for 24 h or 48 h (DPT concentrations: 4, 6, 8 nM, GEF concentration: 1 μM). The cell viability in lung cancer cells decreased in dosedependent chemical that was measured by MTT assay. Data was representative of three independent experiments and expressed as mean ± SD. *p < 0.05 represent statistical difference between control and DPT or GEF test groups. (**B**) HCC827GR cells were seeded in soft agar and treated with DPT or GEF. The colonies were pictured after 2 weeks by light microscope. (**C**) The colonies were counted as the number of rates of apoptotic cells from triplicate independent experiments and the result was mean ± SD of three experiments (*p < 0.05). (**D**-**E**) HCC827GR cells were treated with increasing concentrations of DPT or GEF for 48 h. Annexin V and PI-stained HCC827GR cells were accessed by Muse cell analyzer. The result represented by 4 different populations of cells: live cells (lower left quadrant), early apoptotic cells (lower right quadrant), late apoptotic/dead cells (upper right quadrant) and necrotic/dead cells (upper left quadrant). (**F**) Whole cell lysate was analyzed by Westernblotting with antibodies against cyclin B1, cdc2, p21 and actin. (**G**-**I**) HCC827GR cells were exposed to DPT or GEF for 48 h. Cell cycle progression and sub G1 population of HCC827GR cells following chemical treatment were examined by Muse cell analyzer. Three experiments were conducted independently, and values represent means ± SD (*p < 0.05, compared with control).

To study the effect of DPT treatment on cell growth, the gefitinib-resistant NSCLC cells, HCC827GR were employed. HCC827GR cells were treated with an increasing concentration of DPT or GEF (1 μM) for 24 h and 48 h and MTT assays were performed to examine the effect of DPT on the cell viability (Fig. 3A). The result showed a significant dose- or time-dependent decrease in cell viability. The rates of viability after the treatments with DPT for 24 h were 90.93% ± 4.98%, 70.56% ± 2.20%, and 49.42% ± 2.29% for indicated concentrations, respectively. The colony formation method is mostly used to determine the malignant potential of cells. The inhibitory effects of DPT on HCC827GR cell growth were validated by the soft agar assay, which indicated marked differences in the number of cell colonies however GEF did not show any inhibitory effects (Fig. 3B and 3C). Suppression rates in response to DPT treatment were approximately 74% at 4 nM, 55% at 6 nM, and 41% at 8 nM, respectively. Higher concentrations of DPT further inhibited the growth of anchorage-independent colony formation. We assessed the effect of DPT on colony formation activity, and it was observed that DPT drastically inhibited the clonogenic activity of HCC827GR cells compared to vehicle or GEF control (Fig. 3B and 3C).

### DPT Induces Apoptosis and G2/M Cell Cycle Arrest in NSCLC Cells

To verify whether the cytotoxic effects of DPT on HCC827GR cells were related to apoptosis, Annexin V-stained cell analysis was performed. Treated cells were stained with Annexin V/7-AAD and detected by flow cytometry. DPT dose-dependently increased apoptosis in HCC827GR cells, but no significant increase was observed in GEF treated cells (Figs. 3D and 3E). The rate of total apoptosis (early and late) in the control group was 3.36% in HCC827GR cells, which increased gradually to 28.81% and 44.86% after treatment with 6 and 8 nM of DPT for 48 h, respectively (Figs. 3D and 3E). To investigate whether the apoptosis induction by DPT treatment is due to cell cycle arrest, flow cytometry analysis using propidium iodide (PI) staining was performed and cell cycle distribution of DPT treated HCC827GR cells was identified. Compared to the control cells, the G2/M phase of HCC827GR cells was accumulated by DPT treatment in a concentration-dependent manner, whereas the G0/G1 phase was decreased accordingly (Figs. 3G and 3H). Treatment with DMSO or 6 and 8 nM of DPT induced an increase in the G2/M population to 40.10%, 40.33%, and 50.37%, respectively (Figs. 3G and 3H). Furthermore, treatment with 8 nM DPT led to an increase in the sub-G1 proportion by about 48.37 ± 2.31% (Fig. 3I). Additionally, we measured the expression of cell cycle-related proteins in HCC827GR cells following DPT treatment (Fig. 3F). Treatment with DPT resulted in up-regulation of p21 protein expression and down-regulation of cyclin B1 and cdc2 levels. These results indicate that DPT can lead to G2/M arrest and apoptosis in HCC827GR cells.

### ROS Accumulation Is Responsible for DPT-Induced NSCLC Cell Apoptosis

**Fig. 4:**
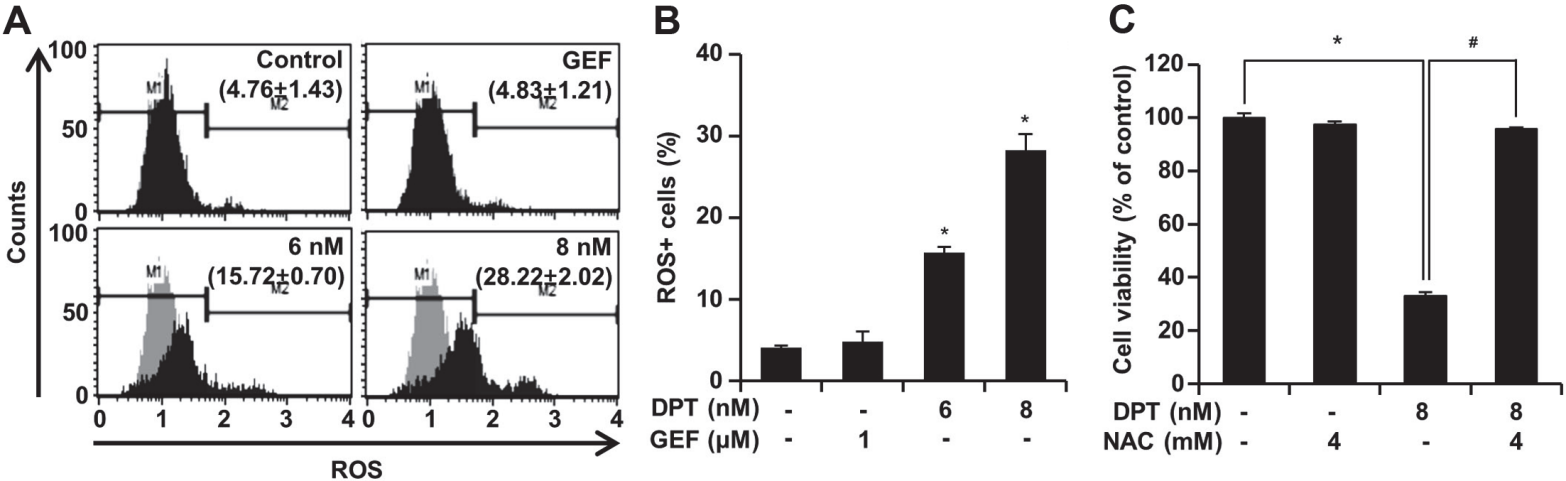
The effect of DPT on Reactive oxygen species (ROS) induction. (**A**-**B**) DPT induces the generation of ROS in HCC827GR cells. The cells were incubated with indicated concentration of DPT or GEF for 48 h and ROS fluorescence was detected by Muse cell analyzer. “M1” and “M2” represent ROS negative or positive population, respectively. (**C**) NAC rescued DPT induced apoptosis through scavenging. Cells were pretreated with 4 mM NAC for 3 h and then treated to 8 μM DPT. Three experiments were conducted independently, and values represent means ± SD (*p < 0.05, compared with control cells, #*p* < 0.05, compared with DPT-treated cells).

High ROS levels are essential for the initiation of apoptosis induced by some anti-cancer agents. The results of this study showed that treatment with DPT led to a rise in ROS generation. The ROS levels in HCC827GR cells were increased by DPT treatment in a concentration-dependent manner (Figs. 4A and 4B). Moreover, the decreased cell viability caused by DPT was strongly blocked by pretreatment with 4 mM of ROS scavenger NAC in HCC827GR cells for 3 h (Fig. 4C). These data indicate that DPT-mediated lung cancer cell apoptosis may be related to intracellular ROS accumulation.

### DPT Regulates MMP and Caspase Activities in HCC827GR Cells

**Fig. 5:**
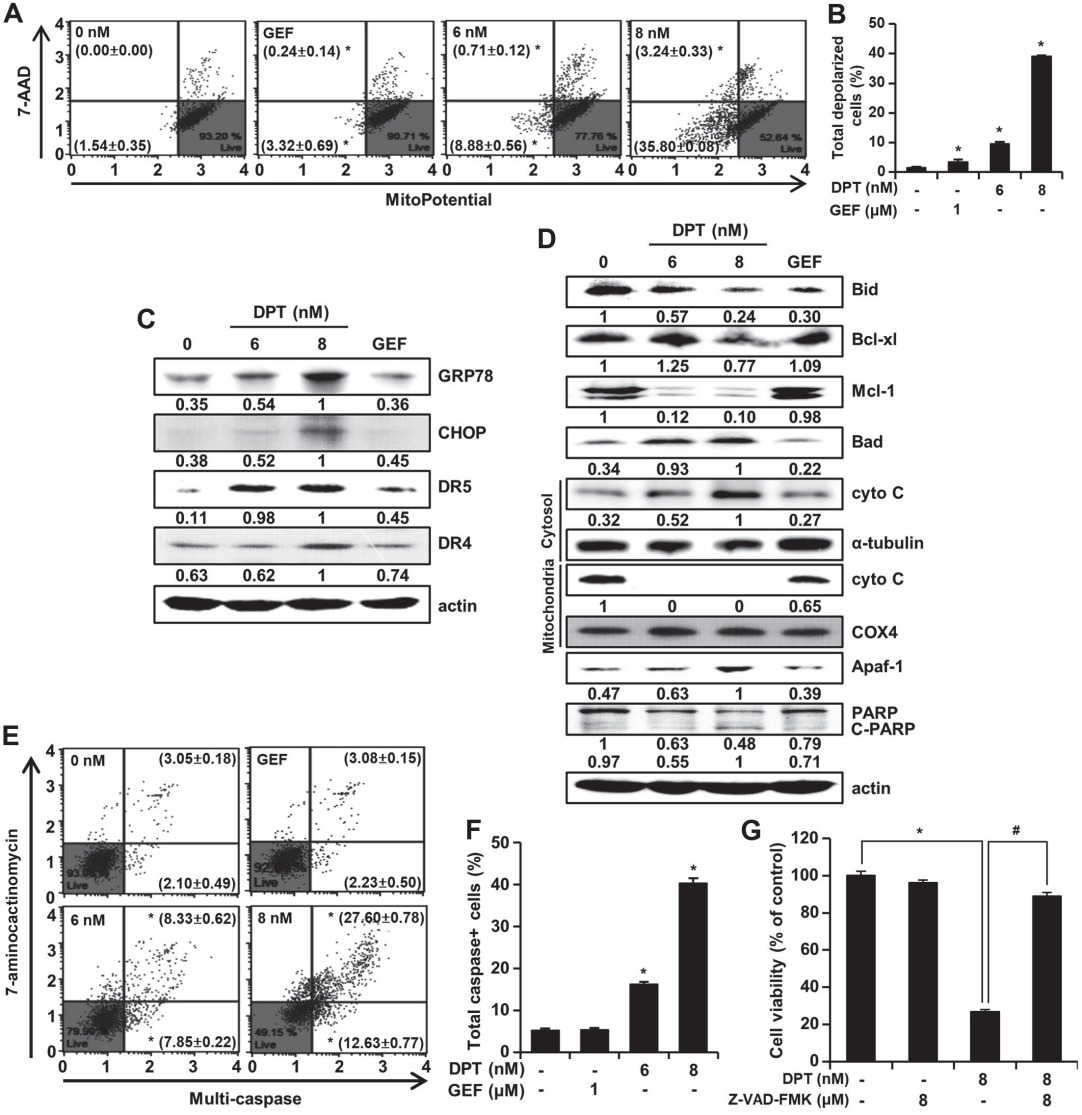
DPT reduces a mitochondrial membrane potential and induces caspase activity in HCC827GR cells. HCC827GR cells were treated with different concentrations of DPT and incubated for 48 h. (**A**-**B**) The movements of fluorescence from right to left indicate depolarization of MMP. The mitochondrial membrane potential was evaluated by Muse MMP kit. Results are expressed as mean ± SD of three independent experiments. (**C**) CHOP, DRs and CHOP proteins were probed in western blotting. (**D**) After treatment of DPT, the proteins were detected by specific antibodies. The expression levels of Bid, Bcl-xl, Mcl-1, Bad, cyto C (cytosol), α-tubulin (cytosol), cyto C (mitochondria), COX 4 (mitochondria), Apaf-1, PARP and cleaved-PARP were normalized to actin. (**E**-**F**) The plots depict the efficacy of DPT and GEF treatments in the lung cancer cell indicated. The cells were treated with DPT at various concentrations or GEF for 24 h. Caspase activity was measured by Muse Cell Analyzer. The data are representative of three experiments independently. (**G**) HCC827GR cells were pretreated with 8 μM of pan-caspase inhibitor that named Z-VAD-FMK for 3h and treated DPT (8 nM) indicated. Statistically significant results are represented as *p < 0.05, remarkably different from DPT-untreated control cells, #*p* < 0.05, remarkably different from DPT-treated cells.

Dysfunction of MMP has been reported to be related to drug-induced cell death and especially, to contribute to inducing apoptosis. Mitochondrial dysfunction is generally decided based on the MMP index detected by flow cytometry. The scattered blots indicating the rate of live, depolarized/live, depolarized/dead, and dead cells are presented in Fig. 5A. In the case of HCC827GR cells, the percentage of live cells decreased from 93.38% to 53.21%, and the percentage of total depolarized cells increased from 1.54% to 39.04% (Figs. 5A and 5B). To further explore the apoptosis mechanism sensitized by DPT, expression levels of GRP78, CHOP, DR5, and DR4 were evaluated by Western blotting. DPT treatment increased the expressions of GRP78, DR5, and DR4 proteins in a dose-dependent manner (Fig. 5C). We performed Western blotting to identify the expression levels of apoptosis-related proteins including Bid, Bcl-xl, Mcl-1, Apaf-1, and PARP. A concentration-dependent decrease in Bid, Bcl-xl, Mcl-1, and PARP expression was observed, while there was a concentration-dependent increase in the levels of Bad, Apaf-1, and C-PARP (Fig. 5D). However, GEF treatment showed the same tendency related to the expression of proteins as control. Activation of the apoptosis pathway can also be related to the release of mitochondrial cyto C. Consequently, we investigated the movement of cyto C from the mitochondrial matrix to the cytosol by Western blotting (Fig. 5D). To further examine the activation of caspase as a necessary step in the apoptotic pathway caused by DPT treatment, the Multi-Caspase Assay was performed. It was observed that 6 or 8 nM of DPT significantly led to the activation of multi-caspases (Figs. 5E and 5F). The percentage of HCC827GR cells undergoing total caspase activity after treatment with 8 nM of DPT was found to be 40.23 ± 1.26%. As compared to the high concentration of DPT, the case of GEF treatment was found to be 5.32 ± 0.43%, similar to the control (Figs. 5E and 5F). Subsequently, we used the caspase inhibitor benzoyloxycarbony (Cbz)-l-Val-Ala-Asp (OMe)-fluoromethylketone (Z-VAD-FMK) to determine whether DPT-induced apoptosis was caspase-independent or caspase-dependent. HCC827GR cells were pretreated with 8 μM of Z-VAD-FMK for 3 h followed by incubation with 4 nM of DPT for an additional 48 h. The results revealed that Z-VAD-FMK significantly reduced the cytotoxicity of DPT in HCC827GR cells (Fig. 5G). This study suggests that DPT-induced apoptosis of HCC827GR cells is mediated through the caspase-cascade pathway.

## Discussion

The currently used anticancer drugs have many side effects including gradual development of resistance to the drugs and in particular, targeted therapeutics are highly resistant [19]. Among the various therapies targeting EGFR, resistance is nevertheless a common phenomenon and remains a challenge [9].

Natural products and their derivatives have been reported to increase the activity of anticancer drugs with fewer side effects. Chinese herbal medicine (CHM) differently called phytochemical has been accepted as complementary and alternative medicine to defeat cancer worldwide. In the treatment of lung cancer or others, CHM has been shown to prolong the survival rate, provide enhanced quality of life, and fewer toxic effects [21]. As a result of screening of natural compounds from various candidate substances, we investigated DPT that reduces the activity of EGFR and MET and conducted necessary research on this substance. In a previous report, GEF sensitivity has been reported to increase when MET was suppressed in gefitinib-resistant HCC827GR cells; therefore, we investigated the phytomedicine that could block both EGFR and MET [22]. Using models of HCC827GR cell line made resistant to GEF, we have examined how DPT affects these cells.

To verify the targeting of EGFR and MET by DPT the pull-down assay with DPT-conjugated beads was carried out employing HCC827GR cell lysate. In vitro and *ex vivo* pull-down assays showed that DPT binds directly to EGFR and MET (Figs. 1B-1D). Through kinase assays, it was confirmed that DPT reduced EGFR and MET kinase activities (Figs. 1E and 1F). For binding to EGFR and MET, DPT inhibited kinase activity by competing with ATP. ATP competition assay (Figs. 1C and 1D) and molecular docking simulation (Fig. 1G) revealed that DPT interacted with the ATP-binding pocket sites of EGFR and MET. Inhibition of EGFR and MET also inhibited the expression of downstream molecules. The results showed that DPT effectively suppressed the phosphorylation of EGFR and MET in HCC827GR cells (Fig. 2).

To demonstrate the effects of DPT on HCC827GR cells, the cell viability experiments with DPT or GEF treatment were conducted. DPT suppressed cell survival by inhibiting EGFR and MET but GEF exhibited no effect on HCC827GR cell viability and proliferation (Figs. 3A-3C). These results are similar to the outcomes of a previous study that states that inhibition of EGFR and MET decreases cancer cell growth in breast squamous carcinoma [23]. Anti-cancer drugs can inhibit cancer cell proliferation by arresting the cell cycle at the G2/M phase [10, 24]. DPT induces apoptosis through G2/M accumulation in the cell cycle and endoplasmic reticulum (ER) stress [25]. Likewise, our data showed that DPT induced G2/M accumulation (Fig. 3H) and ER stress (Fig. 5C). The levels of mitosis-promoting factor cyclin B1 were down-regulated along with up-regulation of cell cycle inhibitor p21, thereby leading to G2/M cell cycle arrest [26].

The accumulation of excessive amounts of ROS are potentially toxic and can lead to apoptosis. ROS was significantly increased at the highest concentration of DPT compared to the control (Fig. 4A and 4B). In addition, NAC and ROS that are processed at the same time induce ROS-dependent apoptosis, thereby suggesting that ROS plays an essential role in regulating cell signal pathway and apoptosis induction in the presence of DPT (Fig. 4C). ROS and ER stress are closely related, and ROS has been proposed as an important component of ER stress-induced apoptosis [27]. GRP78 and CHOP are indicators of ER stress. GRP78 functions as a potential anti-apoptotic factor, whereas CHOP is an important initiating factor of ER stress-related apoptosis [28]. DPT induced ROS formation and ER stress, which are expressed in GRP78, CHOP, DR5, and DR4 biomarkers (Fig. 4 and 5C).

ROS formation is closely related to the mitochondrial pathway. Intracellular ROS formation can lead to irreversible oxidative damage in cellular organelles including mitochondria [29]. Treatment of HCC827GR cells with DPT induced MMP dysfunction and movement of cyto C from mitochondria to cytosol, thereby activating caspases (Figs. 5D and 5E).

In this study, as it was difficult to extract a massive amount of DPT, animal experiments could not be performed. However, in the future study, we would like to perform an extensive investigation related to in vivo validation. According to previously reported papers, DPT showed anti-tumor effects in vitro as well as in vivo [30, 31]. In vitro screening demonstrated that DPT in the nanomolar range (13.95-26.72 nM) was superior to etoposide in several human cancer cell lines [32]. In vitro pharmacodynamics data of DPT for lung cancer cell lines showed that DPT has potent cytotoxic effect in a concentration-dependent manner (maximum effect at 13 nM) [33]. The results of the pharmacokinetic parameters of DPT indicated that DPT was rapidly distributed in rat tissue and the terminal phase half-life was about 90 min [34]. The results of the pharmacokinetic profiles of DPT in lung cancer tumor-bearing mice showed that DPT was rapidly eliminated from plasma with a half-life of about 50 min, and DPT had high affinity between tumor tissues [33]. Taking these results together, DPT can be expected to have a positive effect before entering the clinical phase.

In conclusion, DPT induced cell cycle arrest and caspase-dependent apoptosis by inhibiting EGFR and MET in gefitinib-resistant cells. These results indicate that DPT can exhibit preventive and adjuvant effects in the anti-cancer treatment of NSCLC.
